# The Effect of Dexmedetomidine on the Acute Pain After Cardiothoracic Surgeries: A Systematic Review

**DOI:** 10.21470/1678-9741-2017-0253

**Published:** 2018

**Authors:** Valiollah Habibi, Farshad Hasanzadeh Kiabi, Hassan Sharifi

**Affiliations:** 1 Department of Cardiac Surgery, Faculty of Medicine, Mazandaran University of Medical Sciences, Sari, Iran.; 2 Department of Anesthesiology, Faculty of Medicine, Mazandaran University of Medical Sciences, Sari, Iran.; 3 Department of Medical Surgical Nursing, Faculty of Nursing, Iranshahr University of Medical Sciences, Iranshahr, Iran.

**Keywords:** Pain, Postoperative, Thoracic Surgery, Cardiovascular Surgical Procedures, Thoracotomy, Sternotomy, Adrenergic Alpha-2 Receptor Agonists, Dexmedetomidine, Analgesia

## Abstract

**Introduction:**

Acute post-operative pain remains a troublesome complication of cardiothoracic surgeries. Several randomized controlled trials have examined the efficacy of dexmedetomidine as a single or as an adjuvant agent before, during and after surgery. However, no evidence-based conclusion has been reached regarding the advantages of dexmedetomidine over the other analgesics.

**Objective:**

To review the effect of dexmedetomidine on acute post-thoracotomy/sternotomy pain.

**Methods:**

Medline, SCOPUS, Web of Science, and Cochrane databases were used to search for randomized controlled trials that investigated the analgesia effect of dexmedetomidine on post-thoracotomy/sternotomy pain in adults' patients. The outcomes were postoperative pain intensity or incidence, postoperative analgesia duration, and the number of postoperative analgesic requirements.

**Results:**

From 1789 citations, 12 trials including 804 subjects met the inclusion criteria. Most studies showed that pain score was significantly lower in the dexmedetomidine group up to 24 hours after surgery. Two studies reported the significant lower postoperative analgesia requirements and one study reported the significant lower incidence of acute pain after surgery in dexmedetomidine group. Ten studies found that the total consumption of narcotics was significantly lower in the dexmedetomidine group. The most reported complications of dexmedetomidine were nausea/vomiting, bradycardia and hypotension.

**Conclusion:**

Dexmedetomidine can be used as a safe and efficient analgesic agent for reducing the postoperative pain and analgesic requirements up to 24 hours after cardiothoracic surgeries. However, further well-designed trials are needed to find the optimal dosage, route, time, and duration of dexmedetomidine administration.

**Table:** 

Abbreviations, acronyms & symbols	
CABG	= Coronary artery bypass graft
CTS	= Cardiothoracic surgeries
DEX	= Dexmedetomidine
FDA	= Food and Drug Administration
ICU	= Intensive care unit
NRS	= Numerical rating scales
PCIA	= Patient controlled intravenous analgesia
POP	= Postoperative pain
RCTs	= Randomized controlled trials
SUF	= Sufentanil
VAS	= Visual analogue scale
VRS	= Verbal rating scales

## INTRODUCTION

Acute pain is one of the intense complications after cardiothoracic surgeries (CTS), which can delay patients' recovery and may increase patients' morbidity and mortality^[1]^. Acute pain after CTS has been determined as a main risk factor in the pathogenesis of numerous postoperative side effects such as respiratory failure^[2,3]^. Inadequately controlling the postoperative pain (POP) increase the risk of pulmonary complications due to the diaphragmatic dysfunction and incapability of patients to take large-volume breaths^[4]^. Consequently, effective pain management can play a vital role in reducing patients discomfort and, therefore, it should be a prerequisite for promoting respiratory and cardiac function of patients undergoing CTS^[5,6]^.

In last decades, several pharmacological and nonpharmacological interventions have been developed to reduce acute POP including opioids, paravertebral and epidural infusion of local anesthetics, sedatives, nerve blockades, intrapleural analgesia, nerve stimulation, ketamine, gabapentinoids, selective COX-2 inhibitors, nonsteroidal anti-inflammatory drugs, alpha2- agonists, and aromatherapy^[2,7]^. However, the effectiveness and efficacy of those interventions are variable among studies. Many of those interventions, particularly opioids, have several side effects that can impair cardiac and respiratory function following surgery^[1,2]^. In addition, the benefits of thoracic epidural analgesia as a gold standard for controlling POP have been questioned because of higher risk of severe cardiovascular complications^[8]^. Hence, acute pain management continues to be a challenge in CTS.

Recently, some opioid-sparing analgesics such as dexmedetomidine (DEX) have demonstrated a promising opportunity to decrease the postoperative complications particularly impairment of respiratory function^[9,10]^. DEX has been recommended for sedating agitated patients in the intensive care unit (ICU)^[11]^, because it does not depress the respiratory and cognitive dysfunctions^[9,12]^.

Several randomized controlled trials (RCTs) have examined the efficacy of DEX on POP after CTS. However, a clear advantage of DEX over other analgesics has not been evident so far. Therefore, the aim of this study was to review the effectiveness of DEX for reducing the acute post-thoracotomy/sternotomy pain in comparison with other analgesics.

## METHODS

This systematic review was accomplished in accordance to the PRISMA: the Preferred Reporting Items for Systematic Reviews and Meta-Analyses guidelines^[13]^. Our PICOS research question was formulated as follows: (P) patients undergoing thoracotomy or sternotomy; (I) dexmedetomidine; (C) placebo or other analgesic drug; (O) postoperative pain; (S) trial.

### Eligibility Criteria

Inclusion criteria were: (1) Study designed with RCT; (2) Patients undergoing thoracotomies or sternotomy; (3) Study with at least two groups that compared perioperative (preoperative, intraoperative, or postoperative) administration of DEX with other analgesic agents or placebo; (4) DEX with different routes, dosage, frequency, and duration of administration; (5) POP should be one of the study outcomes.

Conference proceedings, abstracts, letters, and commentaries were excluded. In addition, quasi-randomized trials, nonrandomized trials, studies not published in English and animal trials were excluded.

### Outcomes Measurement

Primary outcomes were (1) POP intensity measured by visual analogue scale (VAS) or verbal or numerical rating scales (VRS or NRS) or POP incidence; (2) number of postoperative narcotic and/or analgesic requirements; (3) postoperative analgesia duration. Secondary outcomes were: (1) number of DEX-associated major adverse events.

### Information Sources

A predefined Medline-based strategy was developed to search the following databases (Appendix 1): Medline via PubMed, SCOPUS, Institute for Scientific Information (ISI) Web of Science, Cochrane Central Register of Controlled Trials, and Cochrane Database of Systematic Reviews. Reference sections of the included trials, published meta-analyses, and pertinent review articles were hand searched to find additional articles.

### Search Strategy

Both subject headings and free-text terms were used in searching the databases. The search strategy contained two components: (1) dexmedetomidine OR adrenergic alpha-2 receptor agonists; (2) pain OR analgesia. These two components were combined using the Boolean operator, "AND", to obtain any link between them. We searched the databases without publication date restriction from the inception of each database until June 12, 2017.

### Study Selection and Data Collection Process

Two authors (FHK-HS) searched the databases using search strategy (n=1789). They independently screened the titles and abstracts of retrieved studies against the predetermined inclusion criteria for selecting relevant articles (1221 title rejected straightaway because of duplicate or irrelevant study. Reasons for excluding an article were documented. The full-text of potentially relevant articles, which met the inclusion criteria, was reviewed for comprehensive assessment against the inclusion criteria. Disagreement about study selection was resolved by discussion and consensus with the third author (VH). In cases that additional data was required, the corresponding author of the study was contacted. Each included study was independently evaluated by three authors (VH-FHK-HS) for content. Then, data extraction table was completed by relevant data of studies that met the inclusion criteria. None of the review authors (VH-FHK-HS) was blinded to reference details during the study selection process.

### Assessing Risk of Bias

The methodological quality of the selected studies was independently evaluated by two authors (VH-FHK) using the Cochrane Collaboration's tool for assessing risk of bias (Table 1). As recommended by tool developer^[26]^, we did not determine the total quality score for each domain, however, in interpreting the results, the limitations of each study were considered.

**Table 1:** Cochrane Collaboration's tool for assessing risk of bias.

Study	Random sequence generation	Allocation concealment	Blinding of		Incomplete outcome data (attrition)	Selective reporting	Other bias
			Participants and personnel	Outcome assessment			
Dong et al.^[14]^, 2017	Unclear	Unclear	Low	Unclear	Low	Low	Unclear
Dutta et al.^[15]^, 2017	Low	Low	Low	Low	Low	Low	Unclear
Jabbary Moghaddam et al.^[16]^, 2016	Low	Unclear	High	Low	High	Low	Unclear
Cai et al.^[17]^, 2016	Low	Low	Low	High	Low	Unclear	Unclear
Priye et al.^[18]^, 2015	Low	Unclear	Unclear	Unclear	Low	Low	Unclear
Ren et al.^[19]^, 2015	Low	Low	Low	Low	Low	Low	Low
Ramsay et al.^[20]^, 2014	Low	Low	Unclear	Unclear	Low	Low	Unclear
Abdel-Meguid^[21]^, 2013	High	High	Unclear	Unclear	Low	Unclear	Unclear
Elhakim et al.^[22]^, 2010	Unclear	Unclear	Low	Unclear	Low	Unclear	Unclear
Ghandi et al.^[23]^, 2005	Unclear	Unclear	Unclear	Unclear	Unclear	Unclear	Unclear
Wahlander et al.^[24]^, 2005	Low	Low	Low	Low	Low	Low	Low
Venn et al.^[25]^, 1999	Unclear	Unclear	Unclear	Unclear	Low	Low	Unclear

## RESULTS

### Study Selection

**Fig. 1:**
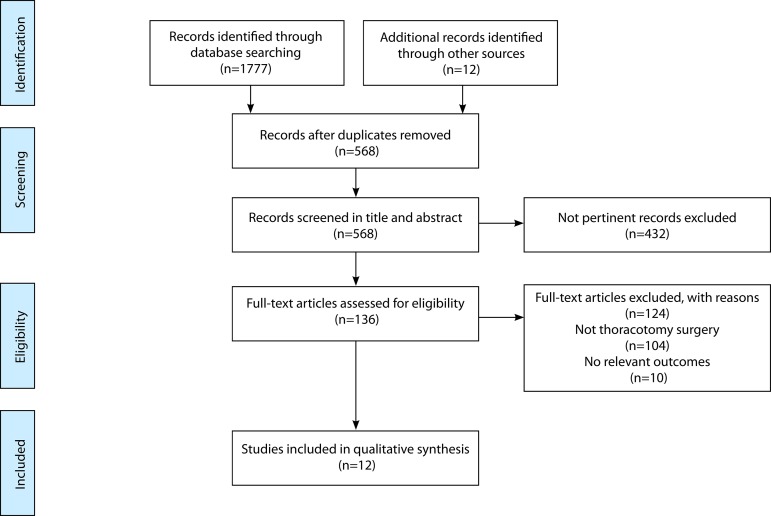
PRISMA flow diagram of search strategy and study selection.

From 1789 citations identified through database searches, 124 articles were examined in more detail. Twelve studies met the inclusion criteria^[14-25]^. The total number of subjects was 804 (DEX, n=419; Control, n=385). Sample sizes ranged from 14 to 54 subjects for each group. The number of patients undergoing general surgery from the total sample size in one study was excluded^[25]^. The flow chart to select the final 12 trials is detailed in Figure 1.

Study Characteristics

All twelve trials were RCT with two parallel groups, except for one study that consist of two groups with different dosages of DEX and a third control group^[19]^. Among the 12 RCTs, the VAS was the most frequently used scale to determine the intensity of POP. Eight studies used the VAS^[14,15,18,20,21,23-25]^, three used the NRS^[16,17,19]^ and one of them used the VRS^[22]^.

Patients' mean age among all trials was 55.89 years (range between 34.4 and 67.7 years). Trials included a total number of 566 (70.4%) male and 228 (28.4%) female, for the last 110 subjects. An error in reported data in one study was found^[14]^ (Table 1) and one study did not report the male/female ratio. Generally, the number of male patients was greater than female.

No statistically significant difference was found between the DEX and control groups regarding the baseline characteristics of patients in all included studies. Table 2 depicts the details for perioperative data and anesthesia techniques.

**Table 2:** Preoperative data, monitoring and anesthesia.

Study	Anesthesia				
	Premedication	Induction	Maintenance	Neuromuscular relaxation	Rescue analgesic use
Dong et al.^[14]^, 2017	MID 0.05 mg/kg/IV 2h preop	0.4 mcg/kg SUF, PROP, SEVO	SUF 1 mcg/kg/h and PROP, SEVO, oxygen, and CIS	CIS	IV injection of meperidine 50 mg
Dutta et al.^[15]^, 2017	Alprazolam on the night before and the morning of surgery	FEN and PROP	ISO and air/oxygen mixture	VECO	MO 3 mg IV
Jabbary Moghaddam et al.^[16]^, 2016	___	FEN 2 µg/kg, MID 0.05 mg/kg, LIDO, and ETO 1 - 2 mg/kg	Oxygen and ISO with 1% - 1.2% end-tidal concentration and FEN	CIS 1 - 2 µg/kg/min	DEX: 0.5 µg/kg/hour Con: MID 0.05 mg/kg
Cai et al.^[17]^, 2016	___	PROP 2 mg/kg, SUF 0.3 mcg/kg	SEVO with a minimal alveolar concentration of 1.0 to 1.3		
	CIS 0.2 mg/kg	Ketorolac 30 mg or tramadol (100 mg)			
Priye et al.^[18]^, 2015	Oral gabapentin 600 mg 45 min before surgery	MID 0.1 mg/kg, FEN 10 meg/kg, PROP 100 mcg/kg/min	___	VECO 0.2 mg/kg	FEN 25 mcg intermittent bolus
Ren et al.^[19]^, 2015	MID 0.5-2 mg	PROP 1.5-2 mg/kg, SUF (TCI 0.2 ng/mL), and CIS 0.2 mg/kg	PROP (TCI 2.4-3.0 mcg/mL), DEX 0.4-0.6 mcg/kg/h) and SUF (TCI 0.2-0.24 ng/mL)	CIS 0.04 mg/kg every hour	30 mg intravenous ketorolac and tramadol (100 mg)
Ramsay et al.^[20]^, 2014	___	PROP, FEN, and SEVO	___	VECO	___
Abdel-Meguid^[21]^, 2013	LOR 2 mg orally one night before surgery and MO 0.1 mg/kg IM 2h Preop	SUF 1-1.5 µg/kg, MID 0.05-0.1 mg/kg	SEVO plus SUF 0.2 mcg/kg/hour, MID 1.5 mcg/kg/hour, and ROCU 0.5 mg/kg/hour	ROCU 0.9 mg/kg	___
Elhakim et al.^[22]^, 2010	MID 0.07 mg/kg IM half an hour before surgery	FEN 3 mg/kg, thiopental 3-5 mg/kg and	End-tidal concentration of 0.3-0.5 vol% ISO	Pancuronium 0.1 mg/kg	Paracetamol
Ghandi et al.^[23]^, 2005	MO 0.1 mg/kg/IM with oral LOR 1 mg	REM 1 µ/kg, MID 0.1 mg/kg and CIS 0.15 mg/kg	REM 0.1 - 0.05 µ/kg/minute, propofol 50 - 75 µg/kg/minute, MID 0.02 - 0.05 µ/kg/minute, CIS and low dose of SEVO	Not reported by authors	Not reported by authors
Wahlander et al.^[24]^, 2005	___	PROP 2 to 3 mg/kg, FEN 2 mcg/kg	Oxygen, nitrous oxide, ISO, FEN, and VECO	VECO 0.1 mg/kg	3 mL 0.125% (3.75 mg) bupivacaine
Venn et al.^[25]^, 1999	___	Remifentanil	MO	___	MID 0.01-0.2 mg/kg/h and MO 2 mg

ABG=atrial blood gas; BIS=bispecteral index; BP=blood pressure; CIS=cisatracurium; DEX=dexmedetomidine; ECG=electrocardiogram; ETO=etomidate; FEN=fentanyl; h=hour; HR=heart rate; ISO=isoflurane; IM=intramuscular; IV=intravenous; LIDO=lidocaine; LOR=lorazepam; MID=midazolam; MO=morphine; NIABP=noninvasive blood pressure; PROP=propofol; REM=remifentanil; ROCU=rocuronium; SEVO=sevoflurane; SpO2=pulse oxygen saturation; SUF=sufentanil; VECO=vecuronium

Types of surgery were thoracic surgery (n=425)^[14,15,17,19,20,22,24]^ and cardiac surgery (n=379)^[16,18,21,23,25]^. As depicted in Table 3, only eight studies including 466 subjects reported their surgeries as elective. Subcategories of thoracotomy operations were esophageal neoplasia resection (n=200), lobectomy (n=77), pneumonectomy (n=26), mediastinal mass or cancer (n=24), pneumothorax (n=7), cyst excision (n=5), decortication (n=4), and bullectomy (n=1), pleurectomy (n=14), other non-categorized major open thoracotomy surgeries (n=67). Subcategories of cardiac surgery were on-pump coronary artery bypass bypass graft (CABG), off-pump CABG, valve surgery, and atrial septal defect closure. We could not determine the exact number of patients in each subcategory of cardiac surgery because some studies did not report the number of patients in each category.

**Table 3:** Characteristics of included trials.

Study	Design	Fixed TRT (drug) for all groups	Postoperative pain outcome assessment and time points	Authors conclusion / DEX reported complications
Dong et al.^[14]^, 2017	Two parallel groups, N= 60 G1: PCIA (n=30) G2: DEX 4 mcg/kg added to PCIA (n=30) Surgery (n of CO, n of DEX): elective major open thoracotomy operations include esophageal neoplasia resection (16, 17), lobectomy (6, 7), pneumonectomy (1, 2), mediastinal mass (2, 2), and pneumothorax (5, 2) INT duration: the first 48h postop period Mean of age: G1=57.3 G2= 55.4, range=32-65 y M/F: 19/31	PCIA program: Drug: SUF 3.0 mcg/kg plus 8mg ondansetron Loading: 20 ml On demand: 2 ml Lock-out: 10 min Background: 4 ml	Outcomes: SUF consumption in the 48h postop period, the mean of pain intensity, the number of PCIA self-administer and meperidine injection Interval: 2, 6, 12, 24, 36 and 48h postop period Scales: VAS	The combination of DEX and SUF in PCIA reduces SUF consumption, the pain intensity, and supplemental analgesic requirements, while maintaining a good hemodynamic stability. DEX reported complications: bradycardia, hypotension, over sedation
Dutta et al.^[15]^, 2017	Two parallel groups, N= 30 G1: Standard drug (n=15) G2: a bolus of DEX 1 mcg/kg over 3 to 5 minutes followed by an infusion of 0.2 mcg/kg/hour added to standard drug (n=15) Surgery (n of CO, n of DEX): elective lung surgeries via anterolateral or posterolateral thoracotomy including lobectomy (2, 8), pneumonectomy (5, 5), cyst excision (5, 0), decortication (2, 2), and bullectomy (1, 0) INT duration: the first 24h postop period Mean of age: G1=34.4 G2= 42.1, range=18-70 M/F: 20/10	Standard drug: A bolus of 15 mL of 0.75% ROPI over 3 to 5 minutes, followed by an infusion of 0.2% ROPI at 0.1 mL/kg/h Thoracic paravertebral block by lignocaine 2% (3 mL) with adrenalin up to 72 hours after surgery	Outcomes: Intraoperative anesthetic drug requirement, pain scores, rescue analgesic use requirement, and incidence of pain syndrome in 2 months Intervals:1, 2, 3, 4, 8, 12, 16, 20, and 24h postop period Scales: VAS	Paravertebral DEX administration is associated with lower number of rescue analgesia, morphine required, total intraoperative fentanyl dose, propofol induction dose, and lower postop pain in 1, 2, 4, and 8 hours. DEX fails to lower the incidence of post-thoracotomy pain syndrome. DEX reported complications: bradycardia, hypotension, over sedation
Jabbary Moghaddam et al.^[16]^, 2016	Two parallel groups; N=104 G1: IV infusion of DEX 0.5 mcg/kg/h (n=50) G2: NS (n=54) Surgery (n of CO, n of DEX): elective CABG INT duration: from the initiation of anesthesia until extubation in the ICU Mean of age: G1=57.3, G2= 55.4 M/F: 71/33	None	Outcomes: the NRS score after surgery and the incidence of postop pain by telephone interview Intervals: two months scale: NRS and BPI	The incidence of postop was significantly lower in the DEX group than that of the control group. Pre-emptive intraoperative DEX may reduce pain DEX reported complications: not reported
Cai et al.^[17]^, 2016	Two parallel groups; N= 94 G1: DEX at a loading dose of 1 mcg/kg for 10 minutes, followed by continuous infusion at 0.5 mcg /kg/h (n=46) G2: NS (n=48) Surgery (n of CO, n of DEX): thoracic surgeries including thoracoscopic lobectomy (12, 10), open thoracic lobectomy (4, 6), incision of esophageal cancer (32, 30) INT duration: the first 48h postop period Mean of age: G1=54.6 G2= 55.4, range=18-65 M/F: 94/0	PCIA program: Drug: SUF 0.8 mg/mL Loading: 2 mL On demand: none Lock-out: 5 min Background: a 4-hour limit of 30 to 40 mL of SUF	Outcomes: The mean of pain at rest and with coughing and dosage of SUF during surgery Intervals: at arrival, 1, 4h, and every 4h thereafter until the 48h postop period Scales: NRS	Intraoperative DEX can reduce the opioid requirement and pain intensity, as well as reduce the cumulative dosage of SUF, NRS at rest, and NRS with coughing scores. DEX reported complications: bradycardia
Priye et al.^[18]^, 2015	Two parallel groups; N= 64 G1: IV infusion of DEX 0.4 mcg/kg/h for 12h without a loading dose, (n=32) G2: NS (n=32) Surgery: elective cardiac surgery using cardiopulmonary bypass (CPB) including coronary artery bypass graft, valve surgery, and atrial septal defect closure. (Note: the number of subjects in each category was not identified by authors) INT duration: Intraoperative until the first 24h postop period Mean of age: G1=41.4 G2= 45.1, range= over 18 M/F: 33/31	None	Outcomes: Postop pain and total fentanyl consumption Intervals: 6, 12, 18, and 24h Scales: VAS	DEX is associated with lower pain score at 0, 6, 12, 18 and 24h postop period and fewer fentanyl consumption than normal saline. Also, DEX could reduce the incidence of delirium. DEX reported complications: without significant complications
Ren et al.^[19]^, 2015	Three parallel groups; N= 125 G1: SUF 0.02 mcg/kg/h, (n=41) G2: SUF 0.02 mcg/kg/h plus DEX 0.02 mcg/kg/h each (n=41) G3: SUF 0.02 mcg/kg/h plus IV infusion of DEX 0.04 mcg/kg/h (n=43) Surgery (n of CO, n of DEX1, 2): thoracic surgeries including thoracotomy in esophageal (one incision) (35, 34, 36) and thoracotomy in cardiac cancer (6, 7, 7) INT duration: the first 72h postop period Mean of age: G1=59.8, G2= 59.9, G3=60.5, range=35-65 M/F: 125/0	PCIA program: Drug: SUF 0.8 mg/mL Loading: 2 mL On demand: none Lock-out: 5 min Background: 2 ml/h, 4h limit of 40 ml Intraoperative DEX: a small bolus of 0.5 mcg/kg then reduced to 0.5 mg/kg/h	Outcomes: cumulative amount of self-administered SUF and the postop pain intensity scores both at rest and with coughing Intervals: 1, 4, 8, 16, 24, 48, and 72h Scales: NRS	Addition of DEX 0.04 mcg/kg/h to SUF improves the analgesic effect of SUF and is associated with greater patient satisfaction without side effects. This combination could decrease the total dosage of SUF during the first 72h after surgery. DEX reported complications: without significant complications
Ramsay et al.^[20]^, 2014	Two parallel groups; N= 38 G1: IV infusion of DEX 0.1 to 0.5 mcg/kg/h, (n=19) G2: NS (n=19) Surgery: major open lateral thoracotomy (Note: sub-categories of thoracotomy has not been identified by authors) INT duration: 18 to 24h postop period Up to 24h after that (42-48h postop) Mean of age: G1=61, G2= 56, range=18-85 M/F: 15/23	PCIA Drug: MO Protocol: not reported, but the two groups were similar in the types of PCA pumps and supplemental opioids used Intraoperative: DEX at 0.2 to 0.5 mcg/kg/h without bolus until 30 minutes prior to transfer to the telemetry unit	Outcomes: the amount of self-administered opioid medication and average pain scores Intervals: 24h after ICU discharge and 24 to 48h postop Scales: VAS	In comparison with normal saline, DEX is associated with lower morphine consumption, however, the mean pain scores between DEX and normal saline groups were similar. DEX reported complications: hypotension, bradycardia
Abdel-Meguid^[21]^, 2013	Two parallel groups; N= 30 G1: DEX at 0.5 mcg/kg/h after the induction of anesthesia, that reduced to 0.3 mcg/kg/h on admission in the ICU and continued for 12h post extubation (n=15) G2: NS (n=15) Surgery: elective coronary artery surgery using OPCAB technique INT duration: The first 12h post extubation Mean of age: G1=55, G2= 52 M/F: 23/7	MO for postop pain management	Outcomes: The median of postop pain and total dose of MO Intervals: 2, 4, 6, 8, 10, 12h postop Scales: VAS	DEX showed a better pain control, a lower consumption of narcotics and earlier extubation time. DEX reported complications: not reported
Elhakim et al.^[22]^, 2010	Two parallel groups; N= 50 G1: DEX 1 mcg/kg plus bupivacaine 0.5% via epidural catheter (n=25) G2: bupivacaine 0.5% via epidural catheter (n=25) Surgery (n of CO, n of DEX): elective open thoracotomy for lung surgery with one-lung ventilation including right lobectomy (12, 11), right pleurectomy (7, 7), and right pneumectomy (6, 7) INT duration: after induction of general anesthesia until the first 24h postop period Mean of age: G1=52, G2=50, range=43-54 M/F: 50/0	Drug: IV crystalloids colloids and fentanyl bolus dose were added to the epidural medication	Outcomes: Postop pain scores, and postop analgesic use requirement Intervals: at 6, 12, 18, and 24h after surgery Scales: VRS	Epidural use of DEX decreases the anesthetic requirements and improves postoperative analgesia as well as shorter the ICU stay DEX reported complications: without significant complications
Ghandi et al.^[23]^, 2005	Two parallel groups; N=100 G1: MO 0.2 mg via PCIA at 4 ml/h (n=50) G2: DEX 0.2 mcg/kg via PCIA (n=50) Surgery: candidates for open cardiac surgery INT duration: after surgery until the first 24h postop Mean of age: G1=66.7, G2= 65.4, range=54-81 M/F: not reported	PCIA, but the protocol not reported by authors	Outcomes: The mean of postop pain intensity and intravenous MO consumption Intervals: 2, 4, 6, 8,10,12, 14, 16 and 18h postop Scale: VAS	DEX is associated with lower pain score in the 2, 4, 6, 8, 10 and 12h postop periods. Male patients experienced lower pain than female in the DEX group. DEX reduced the IV MO consumption during ICU stay. DEX shortened the intubation time. DEX reported complications: without significant complications
Wahlander et al.^[24]^, 2005	Two parallel groups; N= 28 G1: IV loading dose of DEX 0.5 mcg/kg over 20 minutes, followed by continuous IV infusion at 0.4 mcg/kg/h, (n=14) G2: NS (n=14) Surgery: elective thoracotomy for wedge resection, lobectomy, or pneumonectomy (Note: the number of subjects in each category was not identified by authors) INT duration: the first 24h post ICU Mean of age: G1=67.7, G2= 65.7, range= over 18 M/F: 12/16	In the operating room, a TEC loaded using 3 mL of 1.5% lidocaine-epinephrine mixture. PCEA program: Loading: 3 mL 0.125% (3.75 mg) bupivacaine, Lock-out: 20 min Background: 4-hour limit of 30 mL 0.125% (37.5 mg) bupivacaine	Outcomes: need for additional epidural bupivacaine administered by PCEA and the requirement for supplemental opioids (fentanyl) Intervals: at admission to SICU or PACU (zero time point) and then in 1, 2, 3, 4, 12, 16, and 24h postop periods Scales: VAS	IV DEX has beneficial analgesic effects on post-thoracotomy pain when using as an addition to a thoracic epidural infusion of 0.125% bupivacaine. It is unable to decrease the PCEA requirement, but is able to decrease the requirement for opioids and likelihood of respiratory depression DEX reported complications: hypotension and bradycardia
Venn et al.^[25]^, 1999	Two parallel groups; N= 98, cardiac (n=81) G1: IV DEX at a loading dose of 1 mcg/kg over 10 min followed by a maintenance infusion rate of 0.2-0.7 mcg/kg/h, maximum infusion rate of 0.7 mcg/kg/h (n=39) G2: NS (n=42) Surgery: cardiac surgery using cardiopulmonary bypass (n=81) and general surgery (n=17) INT duration: within 1h of arrival on the ICU until the first 24h after that Mean of age: G1=63.3, G2= 64.2, range= over 18 M/F: 54/27	MID 0.01-0.2 mg/kg/h and MO	Outcomes: postoperative analgesia (MO) Intervals: hourly up to first 24h postop period Scales: VAS	Intubated patients receiving DEX required 80% less MID and 50% less MO DEX could reduce the requirements for rescue sedation and analgesia in postop patients for up to 24h. MO requirement was reduced by half in the DEX group. DEX reported complications: bradycardia and hypotension

ABG=arterial blood gas; BP=blood pressure; BPI=brief pain inventory; C=control; CABG=coronary artery bypass graft; DEX=dexmedetomidine; G= group; HR=heart rate; ICU=intensive care unit; INT=Intervention; M/F=male/female number; MO=morphine; NR=not recorded; NRS=numeric rating scale; NS=normal saline; NV=nausea and vomiting; OAA=Observer's Assessment of Alertness/Sedation; OPCAB=off‑pump coronary artery bypass; PCEA=patient-controlled epidural analgesia; PCIA=patient-controlled intravenous analgesia; PNRS: pain number rating scale; Postop=postoperative; Preop=preoperative; RCT=randomized controlled trial; ROPI=ropivacaine; RSS=Ramsey sedation scale; TEC=thoracic epidural catheter; TRT=treatment; VAS=visual analogue scale; VRS=verbal rating score

#### Outcomes Among the Trials

The reviewed trials reported several outcomes. Only the outcomes pertinent to our review were described. The primary or secondary outcomes of included studies were sufentanil (SUF) consumption^[14,17,19]^, the mean of pain intensity^[14,15,17-20,22,23]^, the median of POP^[21]^, the number of patient controlled intravenous analgesia (PCIA) self-administer^[14]^, the number of opioid injection^[14,20]^, morphine consumption^[15,21,23,25]^, the number of intraoperative anesthetic drug requirements^[15]^, fentanyl consumption^[18,24]^, postoperative analgesic requirements^[22]^, need for additional epidural bupivacaine^[24]^, and the incidence of POP^[16]^. The pain was evaluated from one to 72 hours after the operation. Table 3 depicts the additional details for characteristics of included trials.

Table 4 briefly shows a list of the preliminary findings of this review.

**Table 4:** The most relevant preliminary findings of our review.

• DEX is associated with lower postoperative pain scores or incidence after cardiothoracic surgeries in comparison with placebo (normal saline)
• DEX is probability able to reduce the analgesia requirement during and after cardiothoracic surgeries
• DEX is unable to reduce the postoperative pain score or incidence after 36 hours from the start of surgery
• DEX is probability able to improve the postoperative pain control in comparison with morphine
• DEX has noticeable morphine-sparing effects
• DEX could decrease intravenous morphine consumption during ICU stay
• DEX could improve the analgesic effect of sufentanil and decrease the total dosage of sufentanil during the first 24 hours after surgery
• The addition of DEX to epidural bupivacaine could decrease the anesthetic requirements and improve postoperative analgesia
• DEX could decrease the total consumption of narcotics
• DEX could decrease the extubation time
• The most reported complications of DEX were bradycardia, hypotension, and over sedation
• DEX administration is associated with lower risk of respiratory depression
• DEX demonstrated hemodynamic predictability.

Note: Due to the limited number of available trials regarding the effectiveness of DEX, these findings are preliminary; hence, confirmation or rejection of any of these findings warrants further research.

#### The DEX Administration Protocol

Table 5 summarized the protocol of DEX administration among trials. Five studies^[15-17,21,22]^ used DEX in the intraoperative period while seven studies^[14,18-20,23-25]^ used DEX in the postoperative period. Dosage for the intravenous infusion ranged from 0.02 to 0.7 mcg/kg/h and for the epidural catheter was 1.0 mcg/kg^[15,22]^. Two studies added the DEX to the patient-controlled intravenous analgesia (PCIA) pump (dosage ranged from 0.2 to 4 mcg/kg)^[14,23]^. The duration of DEX administration among the trials was varied and ranged from one to 72 hours.

**Table 5:** Protocol for DEX administration in the DEX group.

Study, year	Time and route of injection	Protocol for DEX injection in DEX group
Dong et al.^[14]^, 2017	Start: Postoperatively, after transfer to the general ward End: after 48h; Route: IV using PCIA	A PCIA protocol consists of sufentanil 3 mcg/kg and 8 mg ondansetron was started for all patients. The PCIA was programmed to deliver a 2ml bolus with a lockout interval of 10 min, and a background infusion rate of 4 ml/h. DEX 4 mcg/kg was added to the PCIA for DEX group.
Dutta et al.^[15]^, 2017	Start: Intraoperatively, before induction of anesthesia End: after 72h post-operative period; Route: epidural catheter	All patients received the study medications through paravertebral (multipored epidural) catheter. Patients in the DEX group received 15 mL of 0.75% ropivacaine plus DEX, 1 mg/kg bolus over 3-to-5 minutes followed by an infusion of 0.2% ropivacaine plus 0.2 mg/kg/h of dexmedetomidine at 0.1 mL/kg/h. Paravertebral infusion was stopped and the catheter was removed 72h after surgery.
Jabbary Moghaddam et al.^[16]^, 2016	Start: Intraoperatively, after induction; End: ?h after extubation in ICU; Route: IV infusion	0.5 mcg/kg/h of DEX was infused from the initiation of anesthesia until extubation in the ICU.
Cai et al.^[17]^, 2016	Start: Intraoperatively, before the start of anesthesia End: 30min before the end of surgery; Route: IV infusion	Before anesthesia, patients were administered a loading dose of 1 mg/kg DEX for 10min, followed by continuous infusion at 0.5 mg/kg/h until 30min before the end of surgery.
Priye et al.^[18]^, 2015	Start: Post-operative, after transfer to ICU; End: after 12h; Route: IV infusion	After surgery, patients were transferred intubated and ventilated to the ICU to receive 12h infusion of DEX 0.4 mcg/kg/h without a loading dose.
Ren et al.^[19]^, 2015	Start: Postoperatively, after patients were transferred to PACU; End: after 72h; Route: IV using PCIA	All patient received DEX intraoperatively. After surgery, 2 doses of DEX in addition of sufentanil were compared with sufentanil using same PCIA protocol. PCIA was programmed to deliver a bolus dose of 2 mL, with background infusion of 2 mL/h and a lockout of 5min, 4h limit of 40 mL.
Ramsay et al.^[20]^, 2014	Start: Postoperatively, 18 to 24h after surgery when patients were admitted to the telemetry unit; End: After 24h; Route: IV infusion	An intraoperative infusion of DEX at 0.2 to 0.5 mcg/kg/h was started for all patients that continued during their ICU or PACU. 0.1-0.5 mcg/kg/h DEX was started about 18 to 24h after surgery when patients were admitted to the telemetry unit for up to 24h.
Abdel-Meguid^[21]^, 2013	Start: Intraoperatively, after induction; End: 12h after extubation; Route: IV infusion	DEX started by continuous infusion at 0.5 mcg/kg/h after induction of anesthesia; this was reduced to 0.3 mcg/kg/h on admission to the ICU and continued for 12h post extubation.
Elhakim et al.^[22]^, 2010	Start: Intraoperatively, after induction of general anesthesia; End: after 24h; Route: epidural catheter	The DEX group received DEX 1 mcg/kg in combination with bupivacaine 0.5% 30-40 mg via the thoracic epidural catheter, which was inserted at the T6-7 interspace.
Ghandi et al.^[23]^, 2005	Start: Postoperatively, after transfer to ICU; End: after 24h; Route: IV using PCIA	After transfer of patients to ICU, they received infusion of DEX 0.2 mcg via a PCIA pump in the first 24 hours after surgery.
Wahlander et al.^[24]^, 2005	Start: Postoperatively, on ICU arrival; End: after 24h; Route: IV infusion	The DEX group received an IV loading dose of DEX of 0.5 mcg/kg over 20min, followed by continuous IV infusion at 0.4 mcg/kg/h.
Venn et al.^[25]^, 1999	Start: Postoperatively, after transfer to ICU; End: 6h-24h after extubation; Route: IV infusion	DEX started within 1h of arrival on the ICU with a loading dose of 1 mcg/kg over 10min followed by a maintenance infusion rate of 0.2-0.7 mcg/kg/h to total maximum duration of infusion was 24h.

Note: "?h" means that the end time of medication was not reported by Jabbary Moghaddam.

#### Interventions for Control Group

Different comparators with DEX were placebo (normal saline) in 9 study arms^[14-18,20,21,24,25]^, different dosages of DEX with SUF 0.02 mcg/kg/h in 1 arm^[19]^, morphine in 1 arm^[23]^ and bupivacaine in 1 arm^[22]^.

#### DEX versus Placebo

Nine trials compared DEX with placebo. Intraoperative administration of DEX was compared with placebo (normal saline) in four trials^[15,16,17,21]^, while postoperative administration of DEX was compared with placebo (normal saline) in five trials^[14,18,20,24,25]^. All of these nine trials showed significant lower POP scores in the DEX group. In general, intra- and postoperative administration of DEX could reduce the pain intensity score after surgery in comparison with placebo.

#### DEX versus Morphine

In comparison with morphine (0.2 mg via PCIA), administration of DEX 4 mcg/kg/h via PCIA could improve the pain control during the first 12 hours after surgery and decrease intravenous morphine consumption during ICU stay^[23]^.

#### DEX Addition to Bupivacaine

One study compared the addition of DEX (1 mcg/kg) to epidural bupivacaine 0.5% with epidural bupivacaine 0.5% and found that epidural use of DEX could decrease the anesthetic requirements and improve postoperative analgesia^[22]^.

### Main Outcomes

#### Post-Operative Pain Intensity or Incidence

Nine trials reported the POP scores at different time points^[14,15,17-23]^. Table 6 shows the POP scores at different time points, which were significantly lower in the DEX group. Only one trial^[14]^ showed a significant lower pain intensity 36 hours after surgery in the DEX group. The incidence of POP in the DEX group was significantly lower in the DEX group when DEX was administered intraoperatively via IV route^[16]^. The median of POP was significantly lower at all time points up to 24 hours in DEX group when DEX was administered intraoperatively via IV route^[21]^. The POP scores and morphine consumption were significantly lower in the DEX group when DEX was used intraoperatively via epidural catheter^[15]^. In all of the trials, no significant difference was found between groups 48 and 72 hours after surgery in terms of POP scores. In general, DEX probably is able to reduce the pain intensity score after CTS up to 24 hours.

**Table 6:** Significant lower pain score at different time points after surgery.

Study, year	Time points in hour after surgery												
	1	2	4	6	8	10	12	16	18	24	36	48	72
Dong et al.^[14]^, 2017				*						*	*		
Dutta et al.^[15]^, 2017	*	*	*		*								
Jabbary Moghaddam et al.^[16]^, 2016	The incidence of postoperative pain was reported												
Cai et al.^[17]^, 2016	*		*		*		*	*		*			
Priye et al.^[18]^, 2015	*			*			*	*		*			
Ren et al.^[19]^, 2015	*		*		*			*		*			
Ramsay et al.^[20]^, 2014	*	*	*							*			
Abdel-Meguid^[21]^, 2013	*	*	*	*	*	*	*						
Elhakim et al.^[22]^, 2010				*			*		*	*			
Ghandi et al.^[23]^, 2005		*	*	*	*	*	*						
Wahlander et al.^[24]^, 2005		*	*										
Venn et al.^[25]^, 1999	Pain was not reported												

Note for interpretation:

* Pain score was significantly lower in the DEX group at (the desired hour) after surgery in comparison with the control group.

#### The Post-Operative Narcotics and/or Analgesic Requirements

Ten studies^[14,15,17,19-25]^ found that the total consumption of narcotics was significantly lower in the DEX group. The requirement for postoperative rescue sedation and analgesia in DEX group was significantly lower when DEX was administered postoperatively via intravenous route^[24,25]^. One study compared the addition of different dosage of DEX (0.02 and 0.04 mcg/kg/h) to SUF with SUF 0.02 mcg/kg/h. The addition of DEX 0.04 mcg/kg/h to SUF could improve the analgesic effect of SUF and decrease the total dosage of SUF during the first 72 hours after surgery^[19]^. In general, DEX administration probably is able to reduce the requirements for supplemental narcotic, rescue sedation and analgesia in the postoperative period for up to 24 hours.

#### DEX Adverse Events

Only six trials (n=206) have reported the adverse events of DEX administration. In all of those trials, DEX was administered postoperatively through intravenous injection or using PCIA. As depicted in Table 7, the differences between two groups regarding the adverse events were not statistically significant, except for the occurrence of atelectasis, which was significantly higher in the control group (OR 0.400, CI 95%: 0.177-0.904). Because of incomplete report of some trials, the adverse events rate was not comparable among patients who received DEX intraoperatively and postoperatively. Therefore, the duration and timing of DEX administration (short *vs*. prolonged) on the incident of adverse events was not evaluated.

**Table 7:** The comparison of adverse events between DEX and Control groups.

Adverse Events	In DEX (n)	In Control (n)	Odds ratio (CI 95%)
Atrial fibrillation	10 (53)	6 (56)	1.998 (0.272-14.660)
Bradycardia	7 (62)	4 (62)	1.448 (0.158-13.247)
Hypotension	16 (115)	6 (118)	3.453 (0.714-16.698)
Nausea/Vomiting	59 (208)	80 (213)	0.641 (0.209-1.962)
Pruritus	18 (153)	45 (153)	0.260 (0.068-1.000)
Hypertension	4 (30)	7 (30)	0.505 (0.131-1.951)
Respiratory depression	4 (30)	9 (30)	0.359 (0.096-1.331)
Atelectasis	23 (50)	34 (50)	0.400 (0.177-0.904)
Delirium	8 (41)	12 (41)	0.585 (0.210-1.631)

The most reported complications of DEX were nausea/vomiting^[17,19,20,23,25]^, bradycardia^[14,15,17,25]^ and hypotension^[14,15,20,24,25]^. Two studies did not report DEX complications^[16,21]^ and four studies reported no statistically significant complications between groups^[18,19,22,23]^. One of the included trials reported four events of respiratory depression in the DEX group^[14]^.

### Other Outcomes

The clinical efficacy of DEX on the ICU length of stay was only reported by one study, which showed that ICU stay was significantly shorter in the DEX group than in the control group (2 and 3 days, respectively)^[22]^. DEX efficacy on the time spent on the ventilator was not reported by any of the included trials. In addition, the information regarding the number of patients who admitted to the ICU after surgery and the duration of ICU stay were not clearly reported across the reviewed trials.

### Dealing with Missing Data

In four cases, we contacted the corresponding author to request further information regarding random sequence generation, allocation concealment, additional blinding details, and type of surgery without success and in one case the contact address was not retrievable.

## DISCUSSION

Pain management after CTS is an important issue for clinicians because POP can significantly impair the cardiovascular and respiratory function. The present study, including 12 RCTs, reviewed the effectiveness of DEX in reducing POP. Regardless of the methodological quality of included studies, the overall results are relatively consistent among studies. Approximately all included studies were methodologically homogenous; however, they were different in the sample size, use of analgesic and anesthetic agent, number of measured outcomes, study population, route and timing of DEX administration and type of surgery.

Findings from our review suggest that, compared with normal saline as a placebo, DEX probably is able to reduce the pain intensity score, the number of narcotic consumption and analgesic requirements up to 24 hours. However, due to the low to medium quality of reviewed trials, further studies are warranted to confirm or refute our findings.

Our finding may have noteworthy implications for pain management of adults' patients undergoing CTS, particularly in the first 24 hours after surgery. It is necessary to mention that the use of DEX beyond 24 hours may be associated with a dose-related increase in adverse events and for this reason, the Food and Drug Administration (FDA) has not recommended the use of DEX for more than 24 hours^[27,28]^. However, the safe use of this drug has been reported from 24 hours to more than a week^[28,29]^.

In the present review, the detailed comparison of the results of the included trials was not possible due to differences in intervention protocol and outcomes measurement. Additionally, five of 12 trials^[20-23,25]^ were likely underpowered for the outcomes, since they did not power the sample size. Therefore, the optimal dosage, timing, and route of DEX administration remain to be elucidated in future studies.

Previous studies have revealed that the most effective dosage of DEX for maximum POP reduction is a loading dose of 1 mcg/kg, which is followed by a continuous infusion of 0.5-1 mcg/kg/h^[30]^. In our review, the dosage for the intravenous infusion were ranged from 0.02 to 0.7 mcg/kg/h and only two studies^[17,25]^ infused DEX at maximum POP reduction dose. We also found that a limited number of studies suggested a scientific justification of the rationale for choosing a dose.

The common adverse events of DEX are hypotension at low blood concentrations, hypertension at high blood concentrations, bradycardia and nausea^[29]^. Most of these side effects occur at infusion of 0.2-0.7 mcg/kg/h without a bolus dose^[28,31]^. In our review, the occurrence of respiratory depression was low and reported only in one trials^[14]^, which is consistent with previous studies^[11,28,31]^. Previous study showed that respiratory suppression does not even occur at DEX plasma levels up to 8.0 ng/mL and only there is a risk of over-sedation^[32]^.

DEX possesses analgesic and opioid-sparing effects in the ICU patients^[33]^. DEX, a shorter-acting and highly selective presynaptic alpha-2-receptor agonist, also possesses pharmacologic sedative, hypnotic, anti-anxious, sympatholytic and analgesic properties^[28]^. Its analgesic and opioid-sparing effects are dose-dependent and trigger at spinal cord sites as well as through non-spinal mechanisms^[29]^. It has been suggested that alpha-2A receptors activation, inhibition of the C and A delta fibers signals conduction, and the local release of encephalin are the underlying non-spinal mechanisms of DEX to provide anti-nociception effects^[34]^. In terms of pharmacokinetics, its action starts about 15 minutes after intravenous injection and its peak concentration is achieved within an hour of continuous intravenous infusion. Appropriate pharmacodynamic effects of DEX are revealed between the plasma concentration of 0.5 and 1.2 ng/ml.

Several strategies have been introduced for POP management^[35]^. It is believed that multimodal analgesic approaches combining different analgesic agents with different mechanisms of action can maximize pain relief while minimize the opioid consumption and thus can limit the opioid-induced side effects^[6,36]^. As a method of limiting opioid-induced adverse events, therefore, multimodal POP management has the potential to decrease morbidity and mortality after surgery^[35]^. Consequently, it is expected that the sedative, anesthetic, analgesic, and cardiorespiratory effects of DEX may enhance with concomitant administration with other anesthetic, sedative and analgesic medications^[28]^. In our review, regardless of the route of administration, three studies used the multimodal approaches and found a reduction in the narcotic consumption and supplemental analgesics requirements^[14,17,19]^. In addition, two studies found that the addition of DEX to morphine can reduce the opioid consumption^[20,21]^, the risk of respiratory depression^[20]^ and the time of extubation^[21]^. One study found that epidural use of DEX plus bupivacaine 0.5% plus fentanyl can decrease the anesthetic requirements and provides effective post-operative analgesia^[22]^. It should be noted that the peridural (epidural) form of DEX has not been officially approved by any drug administrations around the world. However, in many clinical practices, the off-label form of DEX has been used in various scenarios in the operating room including thoracic epidural anesthesia, regional anesthesia block, intubation, monitored anesthesia care sedation, cardiothoracic surgery, and neurosurgery. The United States FDA has only approved the form of intravenous injection of DEX^[27]^.

As the findings of our review suggest, several advantages may encourage clinicians to use DEX over other agents for POP reduction. First, DEX does not interfere with respiratory function and has predictable and stable hemodynamic responses. Second, because of its synergistic effects with narcotics and sedatives, DEX can be used to reduce the total dosage of those drugs. Third, DEX has anxiolytic and sedative properties that may improve POP control. Forth, DEX can be used as an adjuvant to local anesthesia; hence, it can improve postoperative analgesia, and reduce the opioid requirement. Fifth, technically, the use of intravenous DEX is easier that paravertebral or peridural route in terms of equipment, skill, and side effects.

These advantages are consistent with the finding of several reviews that have emphasized the analgesic effects of DEX on POP in different sample of patients and surgeries. Schnabel et al.^[30]^ found that the IV administration of DEX compared with placebo or opioids reduces acute POP and opioid consumption, as well as declines the risk of opioid-related adverse events in patients undergoing non-thoracotomy surgeries. Peng et al.^[9]^ found that postoperative PCIA protocols containing opioid-DEX combination have beneficial effects for reducing the POP intensity, postoperative morphine-equivalent consumption and the adverse events. Liu et al.^[37]^ reviewed the efficacy of DEX on perioperative opioid consumption and POP intensity of patients undergoing neurosurgery and found that DEX could reduce opioid consumption and POP intensity. Bellon et al.^[38]^ found that the intraoperative administration of DEX could reduce postoperative opioids consumption and POP intensity in children undergoing surgery.

There are also some studies demonstrating that administration of DEX cannot reduce POP. Jessen Lundorf et al.^[39]^ concluded that perioperative administration of DEX in comparison with placebo seems to have some opioid-sparing effect with no important differences in POP in adult patients undergoing abdominal surgery. Tan and Ho^[40]^ showed that DEX might reduce the length of ICU stay and duration of mechanical ventilation, but increases the risk of bradycardia and hypotension in critically ill adult patients.

### Limitations

This systematic review has some limitations. First, we did not judge regarding the quality of each trials and risk of bias due to the limit number of retrieved trials; however, we used the Cochrane risk assessment tool to demonstrate any risk of bias at each domain. Second, due to the considerable heterogeneity between studies, we could not perform meta-analysis to evaluate statistically the efficacy of DEX over the other analgesic agents. Third, due to the lack of reported data in some of the trials, difference in DEX doses, and different times of administration, we could not synthesis the results based on the subgroups. Forth, the outcome of pain was incompletely measured and reported in some trials; hence, we could not critically appraise the outcome of those trials. Fifth, we cannot compare the DEX group with control group regarding the POP scores based on the type of surgery because the number of patients in subcategories of cardiac surgery was reported incompletely.

## CONCLUSION

In comparison with placebo or other analgesic agents, the use of DEX after CTS is associated with a lower POP intensity, a lower number of post-operative analgesic requirements and a lower number of adverse events, particularly respiratory depression. Thus, DEX can be used as a safe and efficient analgesic agent for reducing the POP up to 24 hours. Overall, data published to date regarding the use of DEX after CTS suggest a marginal clinical benefit. Further well-designed studies with powered sample size are needed to find the optimal dosage, route, time, and duration of administration as well as the best choice of adjuvant analgesia to DEX for reducing POP.

**Table:** 

Authors' roles & responsibilities	
VH	Substantial contributions to the conception or design of the work; or the acquisition, analysis, or interpretation of data for the work; final approval of the version to be published
FHK	Substantial contributions to the conception or design of the work; or the acquisition, analysis, or interpretation of data for the work; final approval of the version to be published
HS	Substantial contributions to the conception or design of the work; or the acquisition, analysis, or interpretation of data for the work; final approval of the version to be published

## APPENDIX A

Keywords for PubMed

("adrenergic alpha-2 receptor agonists"[MeSH Terms] OR dexmedetomidine[Title/Abstract]) AND ("pain"[MeSH Terms] OR pain[Title/Abstract] OR analgesia[Title/Abstract] OR analgesic[Title/Abstract]) AND (Clinical Trial[ptyp] AND "humans"[MeSH Terms] AND English[lang]) AND (Clinical Trial[ptyp] AND "humans"[MeSH Terms] AND English[lang]) =294

SCOPUS (2017-June-15)

TITLE-ABS-KEY ( dexmedetomidine ) OR TITLE-ABS-KEY ( "adrenergic alpha 2 receptor agonist" ) AND TITLE-ABS-KEY ( pain ) AND ( LIMIT-TO ( DOCTYPE , "ar" ) OR LIMIT-TO ( DOCTYPE , "ip" ) ) AND ( LIMIT-TO ( EXACTKEYWORD , "Human" ) OR LIMIT-TO ( EXACTKEYWORD , "Humans" ) ) AND ( LIMIT-TO ( LANGUAGE , "English" ) ) AND ( LIMIT-TO ( SRCTYPE , "j" ) )=701

ISI (2017-June-15)

(TS=(dexmedetomidine AND pain)) AND LANGUAGE: (English) AND DOCUMENT TYPES: (Article)=617

Indexes=SCI-EXPANDED, SSCI, CPCI-S, CPCI-SSH, ESCI Timespan=All years

Cochrane (2017-June-17)

#1 MeSH descriptor: [Pain] explode all trees

#2 MeSH descriptor: [Adrenergic alpha-2 Receptor Agonists] explode all trees

#3 MeSH descriptor: [Dexmedetomidine] explode all trees

#4 (#1 and #2) or (#1 and #4) in Trials= 165
